# Double Tips for In-Plane Polarized Near-Field Microscopy and Spectroscopy

**DOI:** 10.1021/acs.nanolett.4c02826

**Published:** 2024-09-10

**Authors:** Patryk Kusch, José Andrés Arcos Pareja, Aleksei Tsarapkin, Victor Deinhart, Karsten Harbauer, Katja Hoeflich, Stephanie Reich

**Affiliations:** † Freie Universität Berlin, Fachbereich Physik, Berlin, Berlin 14195, Germany; ‡ Ferdinand-Braun-Institut, Leibniz-Institut fuer Hoechstfrequenztechnik (FBH), Berlin, Berlin 12489 Germany; § Institute for Solar Fuels, Helmholtz-Zentrum Berlin fuer Materialien und Energie GmbH, Berlin, Berlin 14109 Germany

**Keywords:** s-SNOM, TERS, TEPL, in-plane excitation, double tips

## Abstract

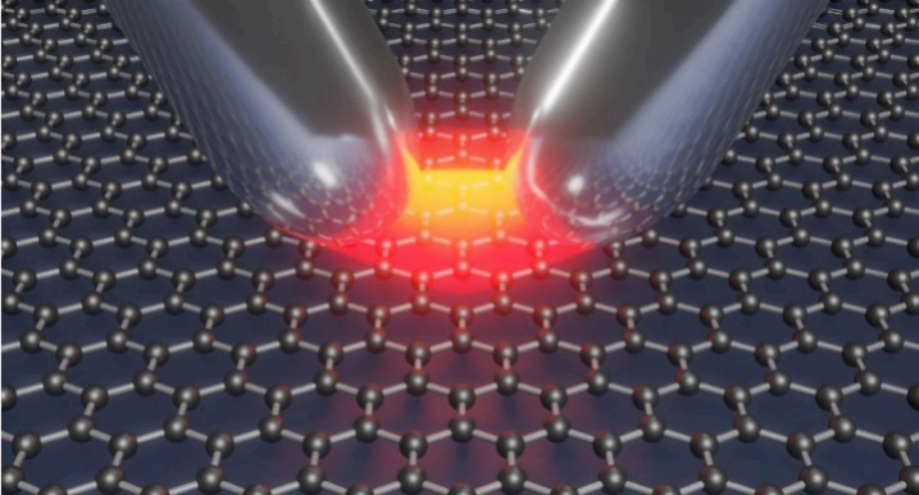

Near-field optical microscopy and spectroscopy provide high-resolution imaging below the diffraction limit, crucial in physics, chemistry, and biology for studying molecules, nanoparticles, and viruses. These techniques use a sharp metallic tip of an atomic force microscope (AFM) to enhance incoming and scattered light by excited near-fields at the tip apex, leading to high sensitivity and a spatial resolution of a few nanometers. However, this restricts the near-field orientation to out-of-plane polarization, limiting optical polarization choices. We introduce double tips that offer in-plane polarization for enhanced imaging and spectroscopy. These double tips provide superior enhancement over single tips, although with a slightly lower spatial resolution (∼30 nm). They enable advanced studies of nanotubes, graphene defects, and transition metal dichalcogenides, benefiting from polarization control. The double tips allow varied polarization in tip-enhanced Raman scattering and selective excitation of transverse-electric and -magnetic polaritons, expanding the range of nanoscale samples that can be studied.

Near-field optical microscopy and spectroscopy exploit localized plasmonic near fields to overcome the diffraction limit of light.^1^ These powerful nanoimaging tools include scattering-type scanning near-field optical microscopy (s-SNOM), tip-enhanced photoluminescence (TEPL), and tip-enhanced Raman scattering (TERS). Scattering-type SNOM is known for its manifold of impressive near-field images, highlighting, e.g., changes in the dielectric function with position and the distribution of doping and strain across a surface. It makes the propagation of surface polaritons directly visible and thereby gives access to the dispersion and losses of the hybridized light-matter excitations.^2−6^ TERS provides chemical fingerprints in nanoscale processes to study chemical reactions at the molecular level and visualize individual molecules with nanometer resolution.^7−20^ The position and distance between localized emitters are measured by TEPL with the potential for ultrahigh resolution bioimaging.^21^

The nanoscale resolution of near-field techniques relies on localized electric fields, that provide enhanced field intensities and high momenta. Both can significantly increase the excitation probability of dipole-carrying matter excitations.^22^ At their core, the localized fields involve the dynamical coupling of electrons in collective motion (i.e., plasmons) with photons.^23,24^ The corresponding quasiparticle is named plasmon-polariton and can be exploited for manipulating light at subwavelength scales, confining electromagnetic fields to nanoscale volumes, and enhancing light absorption and scattering.^25^ In near-field microscopy and spectroscopy sharp metal tips are brought into the proximity of a sample, typically placed within a few nanometers. Plasmon polaritons are excited at the tip by a focused laser beam, creating highly confined electromagnetic near-fields between the tip and the sample. The incoming, elastically (s-SNOM) or inelastically (TERS) scattered light of the probe or its luminescence (TEPL) can be amplified by the near-fieldes.^8,9^ The tip is scanned across the sample to detect the enhanced light as a function of tip position, resulting in an optical image with a resolution that is given by the size of the tip apex.

The enhancement in near-field techniques depends critically on the coupling between the localized field and the sample. The key parameters are the tip–sample distance and the polarization of the plasmonic near-field. In the commonly employed AFM setups, the tip-generated fields are mainly polarized out-of-plane along the *z-axis* (cf. Figure 1A), whereas the dipole transition moments of excitations in two-dimensional materials, large organic molecules, and planar nanostructures are predominantly in-plane.^26^ This misalignment limits tip–sample coupling and thus the tip-enhancement factors.^26^ Controlling the near-filed orientation in scattering-type SNOM would also allow to selectively excite transverse magnetic (TM) and electric (TE) polaritons and waveguided modes, which has been highly challenging for out-of-plane polarization.^27,28^

**Figure 1:**
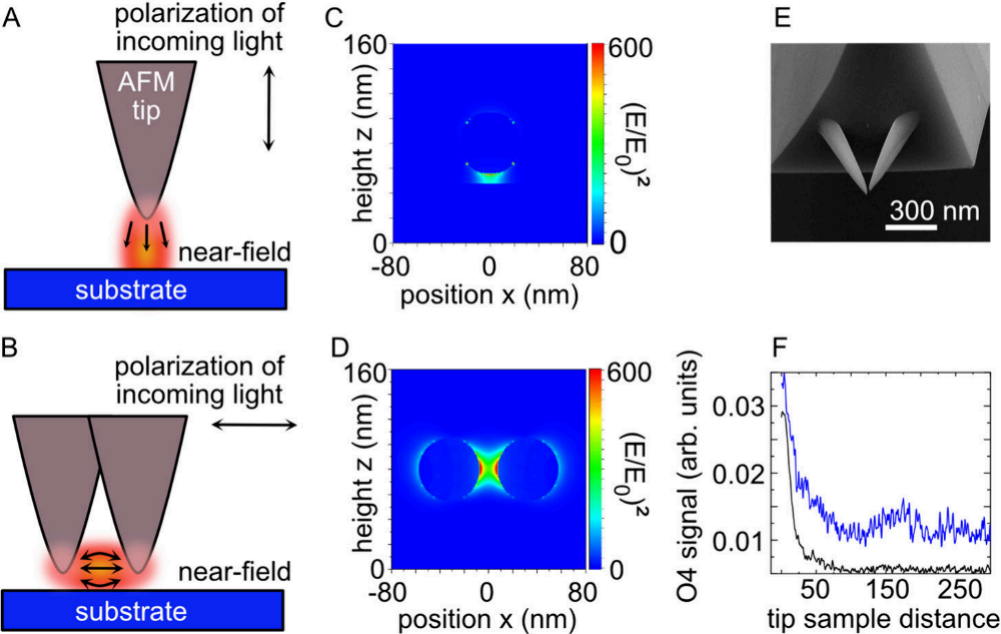
(A) Scheme of a conventional single tip used in TERS and s-SNOM. The polarization of the incident light is p-polarized as indicated. The orientation of the near-field is mainly out-off plane, perpendicular to the substrate. (B) Scheme of a double tip used in this work. (C) Calculated near-field between a single silver particle and a gold substrate (gap-mode configuration). (D) Calculated near-field between two silver nanoparticles that mimic the apex of the double tip. The orientation of the near-field is mainly in-plane, parallel to the substrate (E) SEM image of a double tip grown by FEBID. (F) Amplitude as a function of tip–sample distance demodulated at the fourth harmonic recorded at 480 nm excitation with the double tip (blue, s polarized), and a single silver tip (black, p polarized). Both curves show the expected decay of the amplitude signal within 50 nm as is characteristic for near-fields.

Here we present a metallic double tip for the in-plane excitation in near-field microscopy and spectroscopy. The tip effectively implements scanning with a nanodimer, where the near-field is excited between two metal nanoparticles resulting in in-plane polarization.^29,30^ The double tip comprises two single tips that point toward each other and are grown by direct electron beam writing. As in the nanodimer case, their optical near-fields couple efficiently to in-plane polarized excitations in absorption, light scattering, and luminescence and allows to exploit optical polarization selection rules. The improved enhancement and polarization control is achieved with only a negligible loss in resolution due to the larger extension of the plasmonic hotspot, as we show in a series of near-field microscopy and spectroscopy experiments on graphene, transition metal dichalcogenides (TMDs), and boron nitride nanotubes (BNNTs) supported by finite difference time domain (FDTD).

The excitation with in-plane polarization is realized with two metallic tips with a gap that is much smaller than the tip diameter, Figure 1B. The coupling of the localized surface plasmons of both tips produces a near-field hotspot between them. When illumined with light of 480 nm wavelength polarized parallel to the dimer axis, Figure 1D, a silver dimer is predicted to show a stronger hotspot than a single antenna under out-of-plane polarization. Crucially, the double tip hotspot is polarized mainly in-plane, whereas the single tip yields out-of-plane polarization. For completeness, we add simulation for different dimer configurations (i.e., tip–tip and tip–substrate distances) to the Supporting Information (SI). The simulations demonstrate that positioning a nanodimer close to a gold substrate (5 nm) leads to a reduction in the maximum field enhancement between the nanodimers. Furthermore, this configuration causes an extended near-field, creating a larger hotspot with an extensive near-field interaction between the substrate and the dimer. At the outer extremities, the near-field is repressed, as expected by other simulations in in-plane polarization.^31^ The proposed silver nanodimer shows a plasmon resonance in the visible 420–500 nm, which is ideal for TERS and TEPL as they normally operate in the visible and near-IR spectral range.

While structured AFM tips were reported before to increase field enhancement,^32−36^ their ability for polarization control has not been realized so far. Notably, the first applications of dimer and bow-tie antennas as tips for nanoimaging were demonstrated on single quantum emitters.^34−36^ These tips were specifically designed to maximize near-field enhancement. However, the exciting potential to adjust their polarization to be in-plane was not, being highly interesting for future research.

Double-tips on AFM cantilevers as depicted in Figure 1E were fabricated by focused ion and electron beam processing followed by glancing angle deposition of silver. The focused Ga ion beam was used to mill a well-defined plateau on a conventional AFM cantilever. Subsequently, a focused electron beam was used to locally dissociate a gaseous precursor, a process called focused electron beam-induced deposition (FEBID).^37−39^ The beam path during the direct writing process was carefully optimized for mechanical stability of the antennas and to realize gap sizes of about 50–70 nm that account for the expected thickness of the added silver layer. The final silver-coated dimer antennas had a total length of 5 μm and a gap size of about 50 nm which determines the spatial resolution and field enhancement (cf. methods in SI).

In the following, we demonstrate double tips for near-field imaging and TERS using in-plane polarization. Techniques like TERS and s-SNOM were used to evaluate the performance of the d tips by imaging topographies of 2D samples and nanotubes. Resolution and enhancement were compared between double and single silver tips using the dual s-SNOM operating in tapping mode.

We confirmed the near-field presence between the tip and substrate by recording approach curves at higher harmonics in s-SNOM. Adjusting the parabolic mirror for maximum signal at the fourth harmonic frequency, and when retracting the tip, we observed a strong signal decay within the first 100 nm, indicating the near-field’s extension of some tens of nanometers. Approach curves for both double and single silver tips showed expected behavior. The fourth harmonic near-field amplitude decreases rapidly within 100 nm tip–sample distance, highlighting that a near-field is generated,^40^ and the demodulation on the fourth harmonic efficiently suppresses the optical background signal.

Despite its two-tip character the double tips work for obtaining topography images through the AFM with some decrease in spatial resolution. Scattering-type SNOM and TERS setups deliver AFM topography images simultaneously with the optical microscopy and spectroscopy response. In fact, a reliable AFM system is a key requirement for demodulation in s-SNOM setups.^41^ To demonstrate that the double tips are suitable for recording the nanoscale topography, we imaged an MoS_2_ flake and a boron nitride nanotube (BNNT). The edge of the MoS_2_ flake is well observable when recorded with the conventional cantilever, Figure 2A, and the double tip, Figure 2B. Both images show a second step inside the flake and deliver similar sample height (double: 61 nm, single: 60 nm). We estimate the spatial resolution by scanning over the flake edge, Figure 2E. We estimate the spatial resolution to be 30 nm for the double tip, which is decreased compared to the 5 nm determined for the conventional tip.

**Figure 2:**
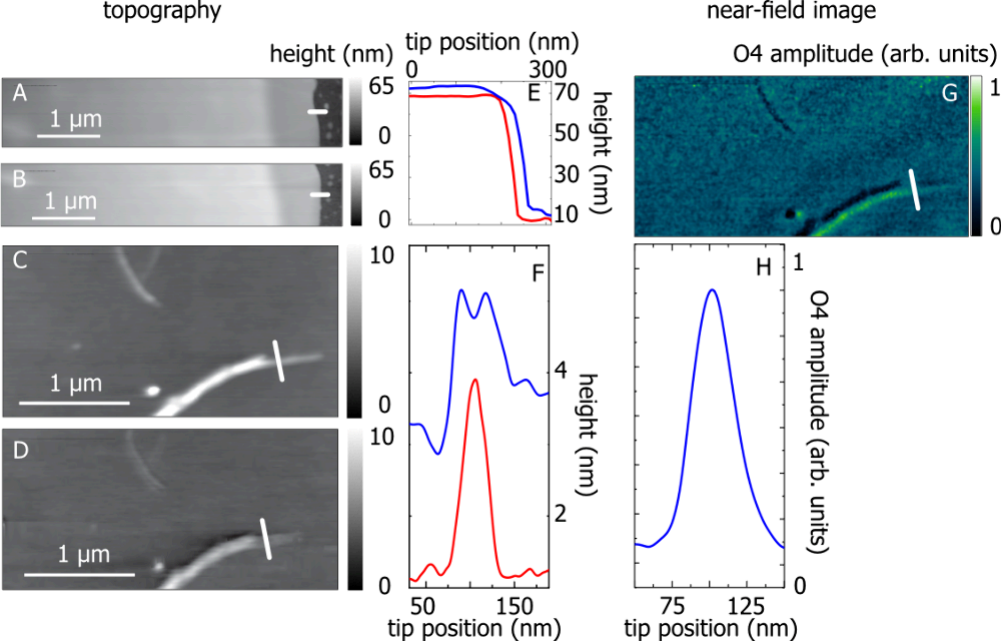
Topography of an MoS_2_ flake taken with (A) a single and (B) a double tip; topography of BNNTs on silicon recorded with (C) a single and (D) a double tip. (E) Cross section extracted from (A) and (B) showing the height of the MoS_2_ flake as a function of tip position, red: single tip, blue: double tip. (F) Cross section of the BNNTs for both tips, same colors as in (E). (G) Optical amplitude image of BNNTs recorded with a double tip in the s-SNOM. (H) Cross section extracted from (G) at the same position as the topography cross-section. The white lines indicate the position where the cross sections were extracted.

With a very narrow nanoscale structure, we can directly observe the splitting of AFM features due to the double tip. The topography of BNNTs recorded with a single tip, Figure 2C, shows a nanotube bundle of 8 nm in height that gets thinner toward the end. At the top of the image, two individual BNNTs are visible. The same topography taken with the double tips, Figure 2D, excellently reproduces the BNNT bundle. The single BNNTs, however, exhibit a double structure. This is clearly shown as two maxima in the height profile of the double tip, blue line in Figure 2F, whereas the conventional tip shows only a single peak (red). Each of the double tips provides the topography information that is averaged in the image. Since the tip spacing (30 nm) is wider than the diameter of the tip apex, we observe the BNNTs twice, an artifact that also occurs with broken AFM tips. However, the optical near-field of the double tips has a single hotspot, Figure 1, so the double image is absent in optical microscopy. The fourth harmonic near-field optical image in Figure 4G was recorded simultaneously with the topography, Figure 3D; it is free of artifacts and shows the BNNTs without any double structure.

**Figure 3:**
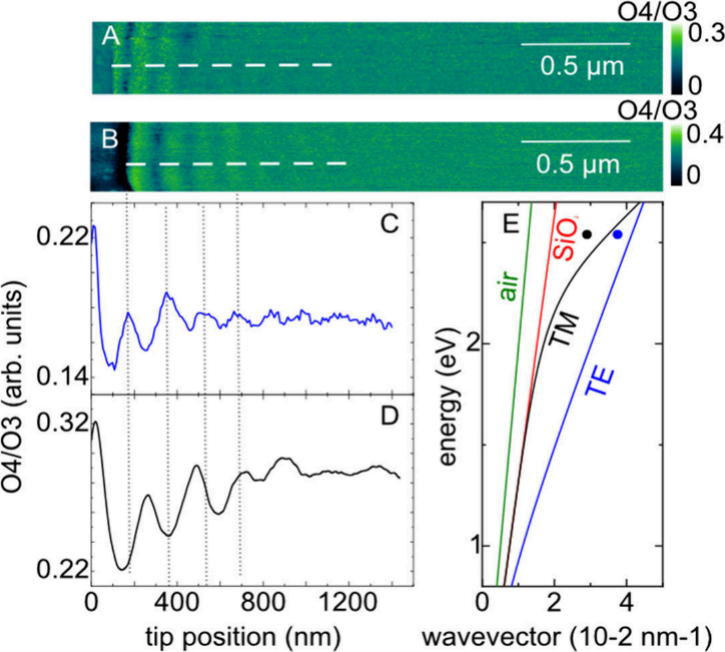
Optical near-field images of few-layer MoS_2_ with (A) a double tip and (B) a single silver tip. (C) and (D) extracted amplitude profiles from panels (A) and (B), respectively. (E) predicted dispersion for TE and TM waveguided modes in MoS_2_ flakes. The blue and black dots are determined from the near-field images in panels (A) and (B). For completeness the light dispersion in air and SiO_2_ are shown.

Double tips allow for finely tuning the polarization direction in scattering-type SNOM by controlling the laser polarization, which has been elusive so far. Two-dimensional flakes support ordinary, transverse electric (TE) and extra-ordinary, transverse magnetic (TM) waveguided modes. These waveguided modes couple to the optically active excitations.^27,42^ In the strong and ultrastrong coupling regime, the materials excitations become of mixed light-matter or polaritonic character, which is sometimes labeled as self-hybridization to distinguish it from the coupling with photons of an external cavity.^43^ Single-tip experiments predominantly image TM-derived polaritons that have a strong polarization component normal to the plane.^42,44^ Transverse-electric modes may also be excited in this configuration, but the much stronger TM modes overlap and often mask the TE polaritons.^45^ On the other hand, only TE modes may be excited with in-plane polarized near fields as provided by double tips.

The dispersions of TE and TM-derived polaritons differ so that polaritons excited with in-plane and out-of-plane polarized near-fields are expected to have different wavelengths at the same frequency.^27,42^ We measured the s-SNOM near-field images of propagating exciton polaritons in an MoS_2_ flake (65 nm in height). Figure 3A shows the exciton-polaritons excited with in-plane polarization by the double tip, whereas Figure 3B is the image recorded under out-of-plane single-tip polarization. The line scans in Figure 3C and D demonstrate the expected difference in polariton wavelength:^27,42^ The single tip excites the TM mode of the MoS_2_ flake with λ = 235 nm at 2.54 eV excitation energy, Figure 3A, whereas we observe the TE mode under double-tip excitation with a shorter wavelength (λ = 170 nm) or longer wavevector, Figure 3B. Double tips may be used to selectively switch from TE to TM modes by changing the near-field polarization. Previous observations of TE polaritons in two-dimensional materials required flakes with ∼100 nm thickness;^27^ double tips will be able to excite the transverse electric excitations down to the thinnest flakes.

In addition to near-field optical microscopy, the dual s-SNOM offers the opportunity to record tip-enhanced PL spectra. We record TEPL spectra as a function of tip position along a line perpendicular to the edge of a WSe_2_ monolayer, Figure 5. The topography and optical near-field image, Figure 4A and B, show bubbles in the monolayer that were produced during the exfoliation and allow identifying the edge of the WSe_2_ flake. TEPL spectra taken on the monolayer, blue circle in Figure 4A, show a 30% increase in luminescence intensity compared to when the tip is on the gold substrate, red circle, see blue and red spectra in Figure 4C. A TEPL scan across the monolayer edge, white line in Figure 4B, shows the drop in TEPL intensity when the double tips move through the edge, Figure 4D. The step-like function allows extracting the spatial resolution of 38 nm. For comparison, we measured the enhancement by a single tip in gap mode, Figure 4E, and found it to be similar to the double tip. The photoluminescence of WSe_2_ can be excited with in-plane and out-of-plane polarized light. The out-of-plane polarization (single tip) is less efficient in the far field, but the inferior polarization configuration is counteracted by the higher enhancement obtained in the gap mode configuration of a single tip on top of a metal substrate resulting in comparable intensities for the double and single tips. The single tip has a higher spatial resolution (∼20 nm, Figure 4E) and, indeed, the gap mode configuration may yield a resolution of 5 nm and less under ideal conditions.

**Figure 4:**
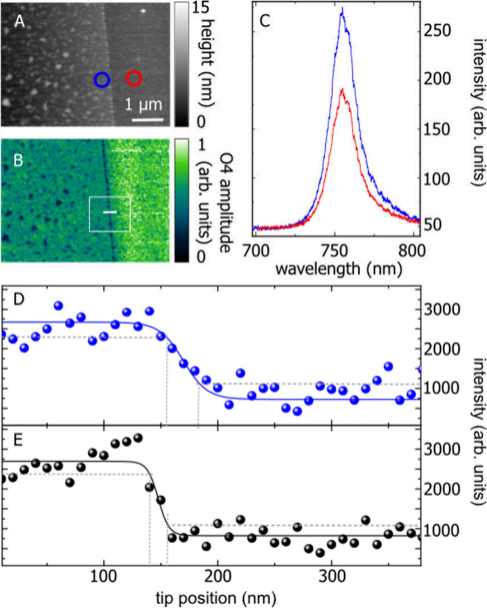
(A) Topography of a WSe_2_ flake on a Au substrate. (B) Corresponding optical near-field image. (C) TEPL spectra recorded when the tip was located on WSe_2_ (blue) and on the Au substrate (red). (D) PL intensity as a function of tip position recorded while the double tip was scanned perpendicular to the edge (white line in (B)). (E) Same as in (D), but with a single tip.

Another near-field technique that greatly benefits from polarization control is tip-enhanced Raman scattering that has strong polarization dependent selection rules.^46−48^ We use graphene to probe the performance of the double tips in TERS because the Raman-active modes of graphene have a constant cross-section and require in-plane polarization of the incoming and scattered light. Many TERS studies have been reported for graphene allowing to benchmark the performance of the double tips.^18,47,48^ We record a topography and a near-field optical image of a graphene sheet, Figure 5A and B, with the dual s-SNOM after which TERS is performed. In both images, the graphene edge is clearly observed including features like bubbles and wrinkles. To determine the TERS enhancement created by the double tips, we recorded TERS spectra of the 2D mode when the tip is close to the edge, spot 1 in Figure 5A, and 30 nm away from the edge, spot 2. The integrated 2D intensity decreases when moving the tip 30 nm away from the graphene, Figure 5C blue and red spectra, indicating resolution and enhancement of the double tip.

**Figure 5:**
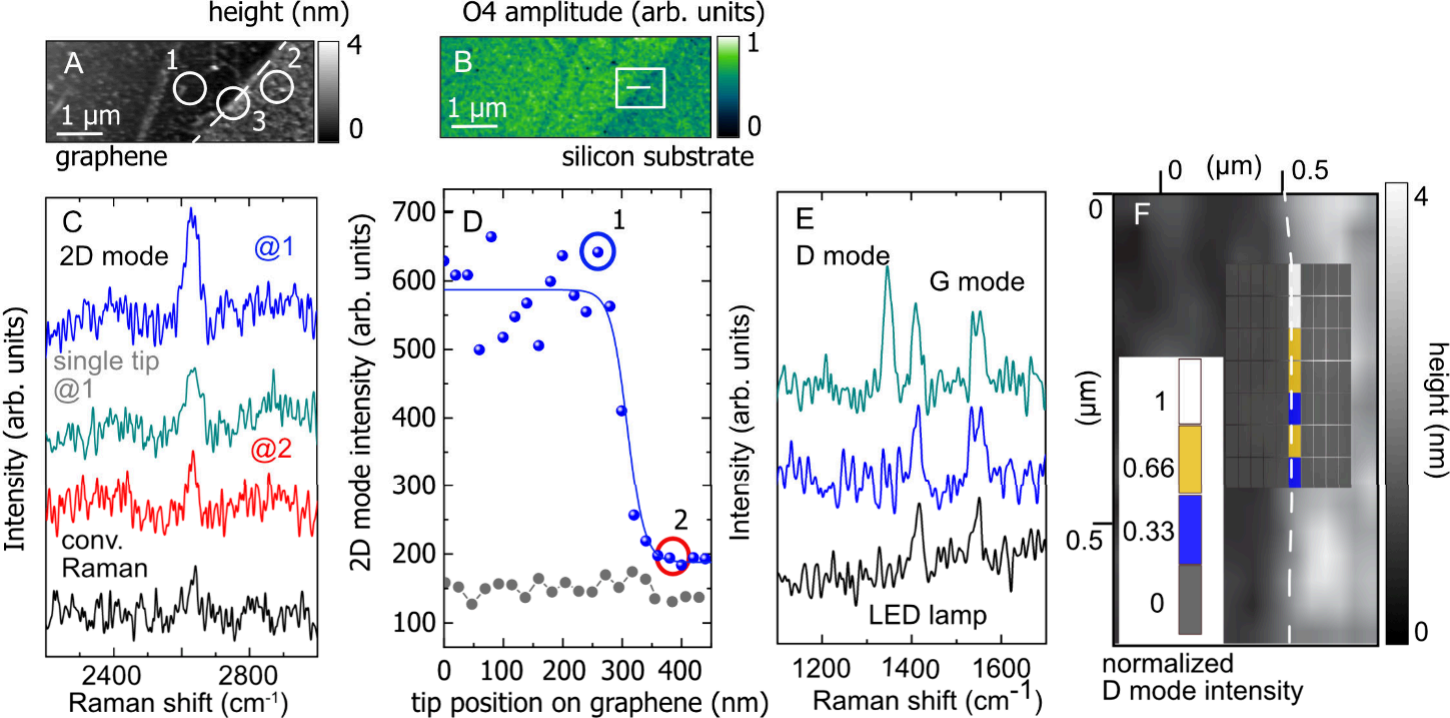
(A) Topography and (B) near-field image of a graphene flake on Si recorded with the double tip. The white line indicates the area where the line scan was recorded. (C) Raman spectra of the 2D mode of graphene recorded at two different positions on the sample with the double tip (blue and red, as indicated in (A)). TERS spectra recorded with a single tip (gray) and without tip (black) are shown for comparison. (D) Raman intensity of the 2D band as a function of tip position for the double (blue) and single (gray) tip. The tips were moved along a line perpendicular to the graphene edge. (E) Raman spectra taken on the substrate (bottom black, position 2 in (A)), the graphene flake (middle, blue @1), and the graphene edge (top gray, @3). The D mode (∼1350 cm^–1^) only appears at the graphene edge; the mode around 1400 cm^–1^ is an LED artifact. (F) Two-dimensional map of the D intensity as a function of double tip position (200 x280 nm^2^ area with 10 × 7-pixel spatial resolution). The D band appears on the edge as determined from the topography image in (A).

It is challenging to quantify TERS enhancement in measurements on 2D materials, because the Raman spectra tend to be dominated by the far-field Raman response.^47^ To estimate the enhancement factor *M* we use the relation
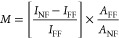
where *I*_NF_ is the integrated TERS and *I*_FF_ the integrated Raman intensity away from the tip, *A*_NF_ = 8 × 10^2^ nm^2^ the area under the tip and A_FF_ = 2 × 10^5^ nm^2^ the area of the laser spot.^32^ By recording the 2D intensity when the tip is on graphene to the intensity measured with the tip on the bare substrate, Figure 5C spectra @1 (blue) and @2 (red), we find *I*_NF_/*I*_FF_ = 3.8. This results in an enhancement factor *M* ∼ 700, an excellent value for TERS experiments. The enhancement for the single tip in out-off plane configuration is 60, i.e., ten times smaller than the double tip. We note that single TERS tips, especially custom-designed pyramid tips or in gap mode configuration, can also achieve TERS enhancement factors on the order of 10^2^,^49−51^ but this required careful optimization in contrast to the proof of concept experiments performed here.

A key application of TERS is chemical-sensitive imaging of surfaces and nanostructures with subwavelength resolution.^52^ To demonstrate the resolution of the double tips in enhanced light scattering, we scan it across a graphene edge while recoding tip-enhanced Raman spectra, Figure 5D. Since the enhancement of the double tips (blue symbols) is higher than for the single tip (gray), the edge is much better detectable with the double tips. From the measured intensities, we extract a resolution of 32 nm for the double tips, blue line fit in Figure 5D, which agrees with the topography scan taken with these tips in Figure 5A and the resolution obtained by TEPL in Figure 4D. The single silver tip has a resolution of less than 20 nm, Figure 5D gray line, but the sensitivity is much lower and the position of the edge is much harder to observe with chemical sensitivity.

To record TERS from a quasi one-dimensional structure with the double tips we measure the intensity of the D band around the edge, Figure 5F. The D mode is activated by defects in graphene, which may be a point defect or a line like an edge.^53−55^ When the double tip is positioned directly at the graphene edge, we observe a strong D band, Figure 5E top spectrum. When retracting the tip or moving it only 20 nm away from the edge the mode disappears, Figure 5E middle and bottom. This highlights the exceptional ability of the double tips to locally excite in-plane polarized Raman processes. The edge of graphene becomes visible as a quasi-one-dimensional defect structure. For reference, we attempted to measure the D mode with a single tip and far-field Raman scattering, but their sensitivity was insufficient to detect any edge-induced scattering.

Finally, 12 d-tips were fabricated for this study, with two successfully utilized for nanoimaging by the dual s-SNOM. The contact AFM mode was not feasible, as the tips were damaged during the approach to the substrate. Future work will focus on developing more robust tip structures exhibiting in-plane polarized near-fields. Preliminary simulations indicate that this can be achieved by cutting a well with a focused ion beam into a single metallic tip with a narrow full width.

In conclusion, we proposed to use AFM double tips to provide in-plane polarized excitation in near-field optical microscopy and tip-enhanced spectroscopy (TERS and TEPL). The double tips were grown free-standing using focused electron beam induced deposition onto conventional AFM cantilevers. We showed that SNOM images taken with such tips allow to control the polarization during excitation leading, for example, to the selective excitation of TE and TM waveguided modes in 2D flakes of transition metal dichalcogenides. The double tip provided an order of magnitude higher enhancement of the cross-section in tip-enhanced Raman scattering on a graphene monolayer, which can be further optimized with smaller gaps and tailored tip design. The increase is due to the orientation of the exciting near field and a better coupling of the double tips to the induced Raman dipole. It comes at the cost of a lower resolution that roughly dropped by a factor of 2 when comparing single and double tips. The in-plane excitation in near-field microscopy and spectroscopy offers a powerful option for scattering-type SNOM, TERS, and TEPL leading to new insights into light-matter interaction and to using optical selection rules in near-field experiments in future.

## Supporting Information Available

Methods including simulations, fabrication, and setup description and calculated near-field for different nanoparticle configurations (PDF)
