# Supplementary material for: Double Tips for In-Plane Polarized Near-Field Microscopy and Spectroscopy
Source: Nano Lett. 2024 Sep 10;24(40):12406–12. doi: 10.1021/acs.nanolett.4c02826 (PMC11468238; doi:10.1021/acs.nanolett.4c02826)
Supplement: Supplementary file 1 — nl4c02826_si_001.pdf [file nl4c02826_si_001.pdf]

Supporting Information

Double tips for in-plane polarized near-field microscopy and spectroscopy

Patryk Kusch[1*], José Andrés Arcos Pareja[1], Aleksei Tsarapkin[2], Victor Deinhart[2], Karsten Harbauer[3], Katja Hoeflich[2] and Stephanie Reich[1]

[1] Freie Universität Berlin, Fachbereich Physik, Berlin, Berlin, 14195, Germany

[2] Ferdinand-Braun-Institut, Leibniz-Institut fuer Hoechstfrequenztechnik (FBH), Berlin, Berlin, 12489 Germany

[3] Institute for Solar Fuels, Helmholtz-Zentrum Berlin fuer Materialien und Energie GmbH, Berlin, Berlin, 14109 Germany

*corresponding author: patryk.kusch@fu-berlin.de

METHODS

Near-field simulations: Silver spherical nanoparticle and nanoparticle dimers were simulated using the finite difference time domain method as implemented in Lumerical FDTD Solutions from Ansys. We use silver nanoparticles with a diameter of d = 50 nm, which was identified as the representative diameter of the tips produced in this work. Silver was described with the dielectric constant measured by Yang et al[1]. We use a mesh override region of 0.5 nm in all directions across the nanostructures. The nanostructures were illuminated from the top with a total-field scattered field source with the electric field polarized along the dimer axis. The electric field was recorded with an electric field monitor. The field enhancement was calculated as the absolute value of the local electric field normalized by the incident field[1].

Fabrication of the double tips: Double tips were produced by electron beam-induced deposition and covered with a thin silver film by glancing angle deposition[2, 3]. The fabrication was performed in a Zeiss Crossbeam 340 dual beam instrument and started from the commercial AFM cantilevers Arrow NCPT. Using focused Ga ions of 30 keV energy and a beam current of 1.5 nA a well-defined flat plateau was milled on the tip. For the following deposition step an electron beam of 15 keV energy and a beam current of about 250 pA was used to locally decompose Pt(η^5-CpMe)Me_3 as the precursor compound. The optimum compromise between mechanical stability and spatial resolution was achieved for a relatively broad antenna radius at the base that decreases towards a tip of very small radius. Therefore, the beam was scanned in spiraling path starting with a base radius of 250 nm that linearly decreased and ends in a single point. The spiral pitch defined the displacement of two consecutive spiral windings resulting a total horizontal length in projection of 2 µm. To account for the decreasing

deposition rate with structure height (which is a consequence of the diffusion-driven precursor supply) the spiral pitch was decreased along the base towards the tip according to a power law with an exponent of 0.5. The beam path was rastered with a constant pixel distance of 0.1 nm and constant dwell time of 200 µs. To realize a quasi-parallel writing with the beam jumping back and forth between the two arms of the dimer the two spiral patterns for the antenna arms were split into 10 equal parts. The gap width in the entire dimer pattern is 125 nm resulting in about 50 - 70 nm gap widths in the deposited structure. The total length of the obtained dimer antenna is about 4 µm with an angle between the antenna arms and the surface of 50°. The optimized pattern is made available as example part of the patterning toolbox FIBomat[4].

The fabricated dimer antennas on the AFM cantilevers were then covered with silver by electron beam evaporation (Scia Vario 100) under glancing angle conditions with a sample tilt of 85° and a rotation speed of 20 rpm and a base pressure of 3.2E-7 mbar. Using a beam current of 180 mA a target thickness of 50 nm was deposited at an evaporation rate of 0.6 nm/s which resulted in about 20 nm of silver layer thickness on the antenna structures.

Near-field experiments: The TERS and s-SNOM experiments were carried out by the dual s-SNOM[5-7]. It is based on a conventional neaSNOM (neaSPEC) that is extended with a spectrometer to detect inelastically scattered light and luminescence. As an excitation source, we use a c-Wave laser (Hübner Photonics) that guarantees wavelength tunable cw excitation in the range 450-650 nm and 900-1300 nm. The setup operates with side illumination, where the laser is focused onto the tip apex by a parabolic mirror at an angle of 30° with respect to the sample. The polarization of the laser in a single tip measurement is parallel to the tip apex (*p*-polarized). The focused light excites plasmons that generate a strongly localized *z*-polarized near-field, i.e., out-of-plane. This near-field enhances both the incoming and emitted light (elastic and inelastic scattering, luminescence). For near-field imaging with the s-SNOM, the elastically backscattered light is detected by a single line silicon CCD. The inelastically scattered light is guided to a spectrometer equipped with a silicon detector (iQuos, Andor) enabling TERS and TEPL. In the SNOM mode, the AFM operates in tapping mode leading to a modulation of the near-field signal. By demodulating the recorded signal on higher harmonics (here the third and fourth harmonic) and by applying the pseudo heterodyne detection scheme we suppress the far-field background in the signal. The recorded signal is split into amplitude and phase, granting access to the absorption and reflection of the near-field optical signal with a resolution down to 20 nm[8]. The tip tapping frequency is 300 kHz and the amplitude 40 nm. To excite a near-field at the double tip we change the polarization of the laser to *s*-polarized

(perpendicular to the tip axis) using a half-wave plate. In this configuration the near-field points from one tip to the other and is oriented parallel to the sample.

Near-field simulations for different configurations

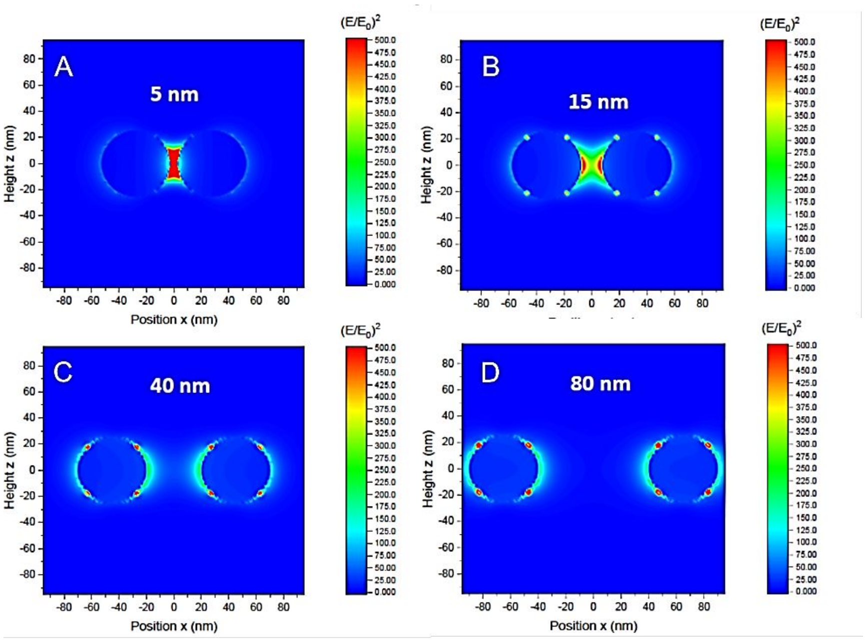

Figure S1 Calculated near-field between two silver nanoparticles with varying gap distance: A 5nm, B 15 nm, C 40 nm, and D 80 nm.

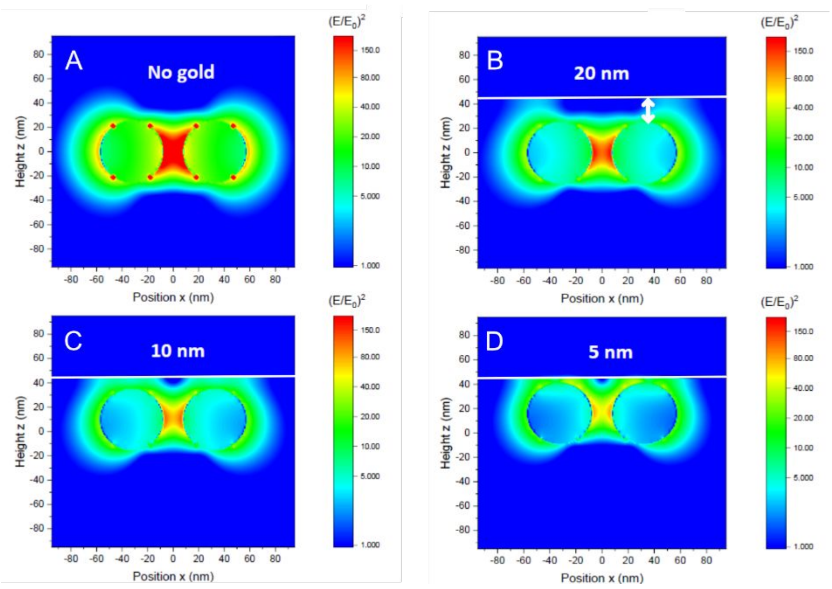

Figure S2 Calculated near-field between two silver nanoparticles with a gap distance of 15 nm and varying distance to a gold substrate. The incoming light is polarized parallel to the dimer axis. In A the gold substrate is not present. In B the distance between dimer and substrate is 20 nm, in C 10 nm, and in D 5 nm. The field intensity is plotted in logarithmic scale.

As shown in previous reports, when a particle is approached to a gold substrate, a gap mode configuration is created where a near-field between the real particle a mirror particle in the metallic substrate is created[9]. When the particle gets closer to the substrate, the maximum near-field intensity increases while its distribution shrinks. This is often used for increasing sensitivity and resolution. A similar behavior can be observed when we vary the distance between the two silver nanoparticles, In Fig. S1A, B, C, D. The simulations show that varying the gap distance of the nanodimer modifies the near-field hotspot properties: when the gap decreases, thus the particles are closer to each other, the maximum field intensity increases and the field distribution shrinks. To study the influence of the gold substrate we calculated the near-field between the dimer without the gold substrate Fig. S2A. Furthermore, we vary the gap distance between the dimer and the gold substrate, as shown in Fig. S2B, C, D. We use a spacing of 5 nm, 10 nm, and 20 nm between the dimer and substrate. To excite the near-field we use in-plane polarization. Interestingly, we find that bringing the silver nanodimer close to the gold causes a more extended near-field, Fig. 2c. However, the maximum field intensity in the gap of the nanodimer decreases. Furthermore, the near-field is squeezed out at the outer extremities of the nanodimer, as expected from previous reports[9].

BIBLIOGRAPHY

(1) Yang, H. U.; D'Archangel, J.; Sundheimer, M. L.; Tucker, E.; Boreman, G. D.; Raschke, M. B. Optical dielectric function of silver. *Physical Review B* **2015**, *91* (23), 235137.
(2) Höflich, K.; Becker, M.; Leuchs, G.; Christiansen, S. Plasmonic dimer antennas for surface enhanced Raman scattering. *Nanotechnology* **2012**, *23* (18), 185303.
(3) Höflich, K.; Feichtner, T.; Hansjürgen, E.; Haverkamp, C.; Kollmann, H.; Lienau, C.; Silies, M. Resonant behavior of a single plasmonic helix. *Optica* **2019**, *6* (9), 1098-1105.
(4) Deinhart, V.; Kern, L.-M.; Kirchhof, J. N.; Juergensen, S.; Sturm, J.; Krauss, E.; Feichtner, T.; Kovalchuk, S.; Schneider, M.; Engel, D. The patterning toolbox FIB-o-mat: Exploiting the full potential of focused helium ions for nanofabrication. *Beilstein journal of nanotechnology* **2021**, *12* (1), 304-318.
(5) Kusch, P.; Mastel, S.; Mueller, N. S.; Morquillas Azpiazu, N.; Heeg, S.; Gorbachev, R.; Schedin, F.; Hubner, U.; Pascual, J. I.; Reich, S.; et al. Dual-Scattering Near-Field Microscope for Correlative Nanoimaging of SERS and Electromagnetic Hotspots. *Nano Lett* **2017**, *17* (4), 2667-2673.
(6) Kusch, P.; Azpiazu, N. M.; Mueller, N. S.; Mastel, S.; Pascual, J. I.; Hillenbrand, R. Combined Tip-Enhanced Raman Spectroscopy and Scattering-Type Scanning Near-Field Optical Microscopy. *J Phys Chem C* **2018**, *122* (28), 16274-16280.
(7) Garrity, O.; Rodriguez, A.; Mueller, N. S.; Frank, O.; Kusch, P. Probing the local dielectric function of WS2 on an Au substrate by near field optical microscopy operating in the visible spectral range. *Applied Surface Science* **2022**, *574*, 151672.
(8) Ocelic, N.; Huber, A.; Hillenbrand, R. Pseudoheterodyne detection for background-free near-field spectroscopy. *Applied Physics Letters* **2006**, *89* (10), 101124-101124.
(9) Kazemi-Zanjani, N.; Vedraine, S.; Lagugné-Labarthet, F. Localized enhancement of electric field in tip-enhanced Raman spectroscopy using radially and linearly polarized light. *Optics Express* **2013**, *21* (21), 25271-25276.
